# Evaluation of radiogenomics for risk stratification of intracranial aneurysms: a pilot study

**DOI:** 10.1007/s00234-025-03702-1

**Published:** 2025-07-15

**Authors:** Sricharan S. Veeturi, Kerry E. Poppenberg, Nandor K. Pinter, Vinay Jaikumar, Elad I. Levy, Adnan H. Siddiqui, Vincent M. Tutino

**Affiliations:** 1 Department of Pathology and Anatomical Sciences, University at Buffalo, State University of New York, Buffalo, USA; 2 Canon Stroke and Vascular Research Center, University at Buffalo, State University of New York, Buffalo, USA; 3 Department of Neurosurgery, University at Buffalo, State University of New York, Buffalo, USA

**Keywords:** Intracranial aneurysms, Aneurysm wall enhancement, Blood transcriptomics, Radiogenomics, Aneurysm risk stratification

## Abstract

**Purpose:**

Aneurysm wall enhancement (AWE) is an imaging biomarker that could aid in risk stratification of intracranial aneurysms (IAs) In this pilot study, we explored the potential of a radiogenomics approach by combining blood-based biomarkers and AWE for better risk stratification of IAs.

**Methods:**

Patient specific vessel wall imaging scans and whole blood samples were obtained, and IAs were classified as high-risk or low-risk using two different metrics: symptomatic status (3 symptomatic vs. 13 asymptomatic) and PHASES score (4 with a high score vs. 12 with a low score). Radiomics features (RFs) were extracted from the pre- and post-contrast MRI for all IA sac walls, and significantly different RFs were identified through univariate analysis. RNA sequencing from whole blood samples for these patients was also performed to identify differentially expressed genes (DEGs) between high and low-risk IA groups. Principal component analysis (PCA) and clustering analysis were applied, using both risk metrics, to evaluate discriminatory power. Lastly, ontological and correlation analyses were carried out to investigate biological mechanisms associated with the DEGs.

**Results:**

Our analysis of 16 IAs identified 12 RFs and 97 genes that were significantly different between symptomatic and asymptomatic IAs (RF: p-value < 0.05; DEG: fold-change > 2, p-value < 0.01). Examining risk with respect to PHASES score, we identified 6 significant radiomics features and 38 differentially expressed genes. Through principal component analysis and clustering analysis, we found that DEGs only and radiogenomics features produced a better separation between high- and low-risk than RFs alone for both risk metrics. Furthermore, we found a significant correlation between 7 unique RFs and 38 DEGs.

**Conclusion:**

We demonstrated that a radiogenomics approach can help in better risk stratification of IAs.

**Supplementary Information:**

The online version contains supplementary material available at 10.1007/s00234-025-03702-1.

## Introduction

Intracranial aneurysms (IAs) are cerebral outpouchings that have high mortality and morbidity rates if they rupture [1, 2]. Accurate and timely risk stratification is paramount for management of IAs [3]. Current size-based risk assessment of IA is not ideal as 50% of all ruptured IAs are < 5mm [4]. To this end, aneurysm wall enhancement (AWE) has emerged as a potential imaging biomarker to identify high-risk IAs [5, 6]. Indeed, studies have shown that IAs exhibiting AWE have a higher degree of local wall inflammation, presence of vasa vasorum and neovascularization, which are indicative of IA instability and rupture [7–9]. However, longitudinal studies have shown that although AWE has a high sensitivity in identifying growing IAs, it has low specificity [10]. A recent meta-analysis by Larson et al. has shown that AWE has a negative predictive value of 96% but a positive predictive value of just 14.4%^11^. Hence, combining AWE with biomarkers of different modalities can help improve its performance in risk stratification.

“Radiogenomics” is a potential approach to improving diagnostic performance of biomarkers by combining radiomics and genomics [12]. The core philosophy of radiogenomics is to fuse genomics that can give molecular level information with image-based radiomics that reflect local pathobiological manifestation of the disease [13]. Recent studies have implemented radiogenomics pipelines for disease prognosis and risk stratification particularly in oncological studies. For example, a study by Chaddad et al., reported a higher performance when using a radiogenomics approach for prediction of outcome of IDH1 wild-type glioblastoma patients [14]. Similarly, another study by Zeng et al., used radiogenomics to predict overall survival of clear cell renal cell carcinoma where they observed that the predictive performance of the multi-omics model improved as compared to traditional radiomics model [15]. These studies underscore the utility of using a combined multi-modal approach as compared to a unimodal analysis for clinical risk stratification.

In this study, we aimed to explore the potential utility of radiogenomics in risk stratification of a small cohort of IAs. To this end, we combined radiomics features obtained using a previously developed pipeline for AWE quantification with transcriptomic data obtained through whole blood RNA sequencing. Two different risk metrics were used to define high risk IAs. We then evaluated the performance of radiomics alone, genomics alone, and their combination in identifying high-risk IAs.

## Methods

### Patient population and aneurysm characteristics

This study was approved by the Human Research Institutional Review Board at the University at Buffalo (STUDY030474433). Blood samples and vessel wall MRI scans were collected from patients undergoing angiographic imaging at Gates Vascular Institute (Buffalo, NY) between 2021 and 2023 as described previously [16]. Only saccular IAs larger than 2 mm in size that had both pre- and post-contrast imaging were included in the analysis. We further excluded dissecting IAs, fusiform IAs, those that exhibited acquisition artifacts, and IAs located in the cavernous segment of the internal carotid artery (ICA) [17]. In patients with multiple IAs, only the symptomatic IA as noted in the electronic health record was included for further analysis. Demographics and comorbidities were obtained from the patients’ medical records. IAs were categorized into high-risk or low-risk aneurysms based on: (1) based on the symptoms of the patient at the time of blood collection, and (2) based on the PHASES score. Patients exhibiting severe headache or blurry vision within two weeks from presentation were classified as symptomatic patients [18]. Additionally, we computed PHASES for each IA as a secondary risk metric, and IAs having a PHASES ≥ 6 were classified as high-risk IAs [19]. The size and size ratio for all IAs was calculated using an in-house MATLAB code as previously described [20].

### Image acquisition and radiomics

Patient specific vessel wall imaging was done as described previously [21]. Briefly, a 3 T MRI scanner (Ingenia Elition 3.0 X, Philips healthcare) was used to acquire a time-of-flight, pre-contrast 3D T1, and post-contrast 3D T1 scans five minutes after the administration of contrast gadolinium (Gadobutrol, 0.1 mL/kg). Anonymized DICOM files were then imported into 3D Slicer to generate 3D segmentations of the IA wall [6]. Radiomics features were extracted from the aneurysm wall in T1-pre and T1-post-contrast using pyradiomics package as previously described [6, 22]. The parameter file used for feature extraction is publicly available on GitHub (pyRadiomics_Aneurysm-extraction). Extracted RFs included shape-based features, first-order features, and textural features [23]. Additionally, difference between the first order features from the pre- and post-contrast MRI were also computed to use as features. A total of 293 RFs per case were extracted.

### RNA sequencing and analysis

**Fig. 1:**
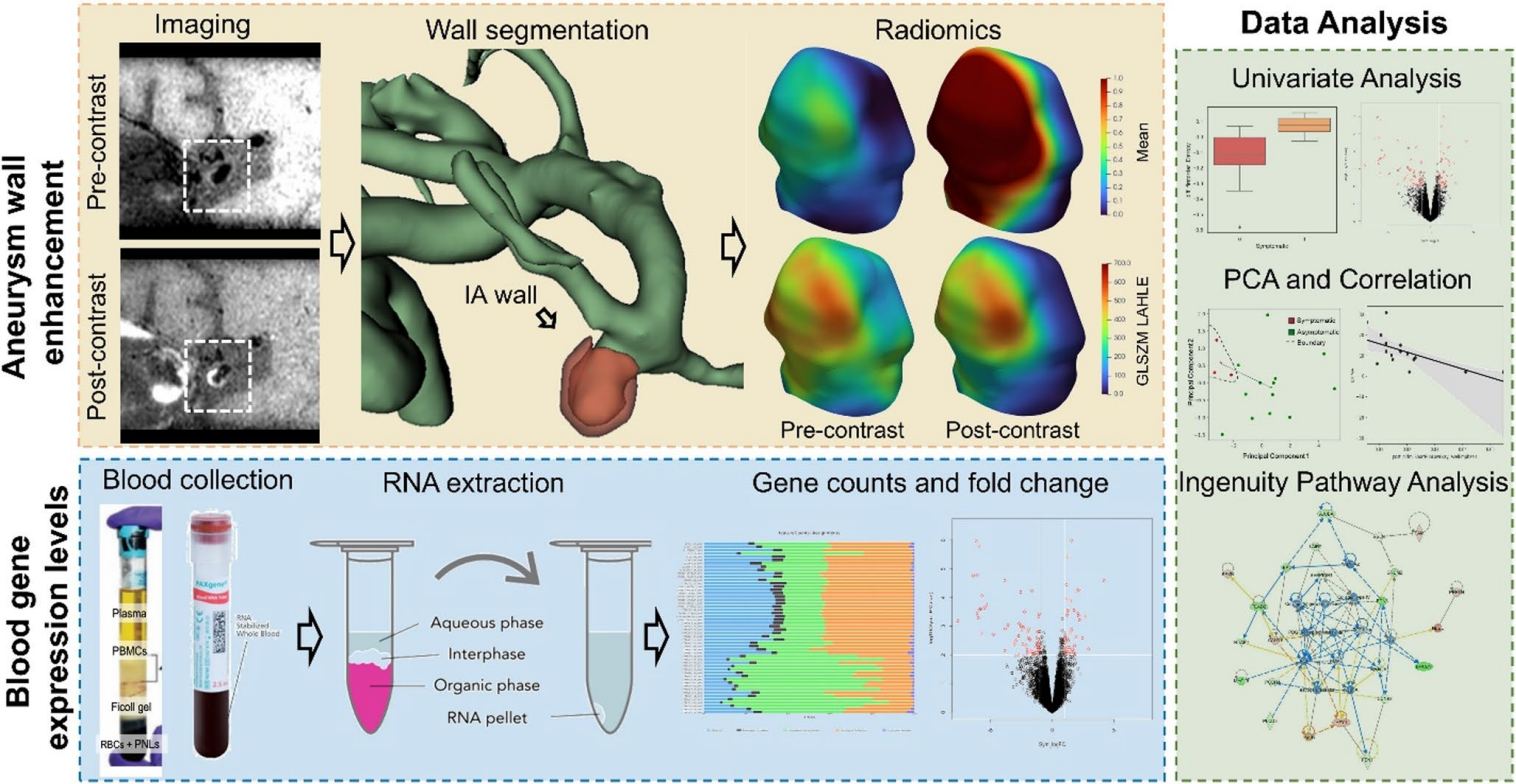
Workflow for analysis of AWE and RNA sequencing. Pre- and post-contrast MRI are registered onto each other, and we extracted radiomics. We also collected blood samples from the same patients and performed gene expression analysis after RNA extraction. We used both these modalities to study differences in radiomics, genomics, and radiogenomics in high and low-risk aneurysms

For sequencing, RNA libraries were prepared using the Illumina TruSeq stranded total RNA gold kit (Illumina, San Diego, CA). All samples underwent RNAseq on the Illumina NovaSeq6000 or the HiSeq2500 System (Illumina) in a series of two batches. Samples were demultiplexed with Bcl2Fastq. Per-cycle basecall files generated by the NovaSeq6000 were converted to pre-read FASTQ files using bclfastq version 2.20.0.422 using default parameters. The quality of the sequencing was reviewed using FastQC v.0.11.5. Potential contamination detected using FastQ Screen v.0.11.1. Genomic alignments were performed using HISAT2 v.2.1.0 using default parameters. National Center for Biotechnology Information (NCBI) reference GRCh38 was used for the reference genome and gene annotation set. Sequence alignments were compressed and sorted into binary alignment map files using samtools v.1.3. Mapped reads for genomic features were counted using Subread featureCounts v.1.6.2 using the parameters ‘-s’ 2, ‘-g’ gene_id, ‘-t’ exon, ‘-Q’ 60; the annotation file specified with ‘-a’ was the NCBI GRCh38 reference from Illumina iGenomes. The entire pipeline for the current study is shown in Fig. 1.

### Radiogenomics analysis

For imaging features, we performed univariate analysis to identify significantly different RFs between high-risk and low-risk IAs, using the symptomatic status and PHASES score. Due to the low sample size, RFs were tested using the non-parametric Mann-Whitney U test. To account for multiple testing, we performed false discovery rate (FDR) correction using Benjamini Hochberg correction to compute modified p-value (q-value). Additionally, to quantify the effect size, we computed the Hedge’s G with bias correction for all the RFs. All the statistical analysis was performed using SciPy package in python [24].

For gene expression features, we conducted differential expression analysis of the RNA sequencing data using R. We filtered dataset to transcripts with expression > 0 in at least 50% of the samples and those corresponding to protein coding genes. This reduced dataset was used with the edgeR package to identify differentially expressed genes (DEGs) between symptomatic and asymptomatic IAs. Differential expression was tested using a generalized linear model. Genes with a p-value < 0.01 and a fold-change > 2 were considered significant. For the selected genes, we computed the q-value after Benjamini Hochberg correction and also computed the effect size using Hedge’s G. This process was repeated for the PHASES analysis. We further examined biological processes and molecular functions associated with the down and up regulated DEGs for both symptomatic and PHASES analysis using gProfiler, considering those with a p-value < 0.05. We also investigated biological significance of the DEGs with Ingenuity Pathway Analysis (IPA) software. Pathways with an|z-score| ≥ 1 were considered significant. Upstream regulators with an|z-score| ≥ 2 were predicted to be inhibited/activated. IPA was also used to construct networks based on gene interactions with the DEGs. A network with a p-score ≥ 21 was considered significant.

We evaluated collinearity between all the significantly different RFs and the normalized gene expression levels of the DEGs for both risk metrics, eliminating all the features that had a high correlation (Pearson correlation coefficient > 0.9). To visualize how these features separated high and low-risk IA cases, we performed principal component analysis (PCA) and hierarchical clustering analysis using the final set of non-collinear significantly different RFs alone, DEGs alone, and a combination of both. To quantify the effectiveness of the PCA using all three modalities of information, we computed the inter-cluster distance as well as the Silhouette score [25]. We first normalized all the RFs and DEGs by removing the mean of each of the features and using unit standard deviation and then computed the principal components and the explained variance of each component using SciKit Learn packages [26]. We then performed hierarchical clustering of the RFs and the DEGs using the online tool Morpheus from Broad Institute (https://software.broadinstitute.org/morpheus). We performed z-score normalization of all the features and used 1-PCC as the metric for hierarchical clustering. Finally, we evaluated potential correlations between the significantly different RFs and DEGs using the Pearson correlation coefficient and the Wald’s test for both risk metrics.

## Results

### Patient population and aneurysm characteristics

Our cohort consisted of 16 patients with IAs of which 3 were symptomatic and 4 had a PHASES score ≥ 6, which we considered high-risk. Through univariate analysis (Mann-Whitney for continuous features and Chi-squared for binomial features) of patient information and aneurysm characteristics, we observed that symptomatic patients were younger, had smaller IAs, and IAs were mostly located in the posterior circulation or at the anterior communicating artery (AComm), although none of these were significantly different. Conversely, we observed that high-risk patients as defined by the PHASES score were older and had larger IAs that were located in the middle cerebral artery and the posterior circulation (Supplementary Table 1). The IAs in the high-risk group also had a statistically significantly higher aspect ratio (*p* = 0.039).

### Radiomics analysis between high-risk and low-risk aneurysms

A total of 12 RFs were significantly different between symptomatic and asymptomatic IAs (Supplementary Table 2) with Hedge’s G effect size ranging from 0.820 to 1.657. Of these, 4 were derived from post-contrast MRI, and 8 were from the difference between pre- and post-contrast MRI. We observed that the symptomatic IAs had a lower Gray Level Dependence Matrix (GLDM) small dependence emphasis, and Gray Level Size Zone Matrix (GLSZM) small area emphasis in post-contrast MRI. Additionally, they had a higher GLSZM size zone non-uniformity, GLSZM zone%, and Neighboring Gray Tone Difference Matrix (NGTDM) complexity in the difference between pre- and post-contrast MRI.

In the PHASES based risk assessment, we observed that there were 6 significantly different radiomics features, all of which were based on the difference between pre- and post-contrast MRI with Hedge’s G effect size ranging from 0.33 to 1.56. High-risk IAs were characterized by a lower cluster prominence, and a higher gray level run length matrix (GLRLM) and GLSZM non-uniformity (Supplementary Table 3) in the difference between post and pre-contrast radiomics. There were no common significant RFs between the two classifications (symptomatic status and PHASES) examined as there were no common cases between both risk metrics.

### Differential expression analysis between high-risk and low-risk aneurysms

**Fig. 2:**
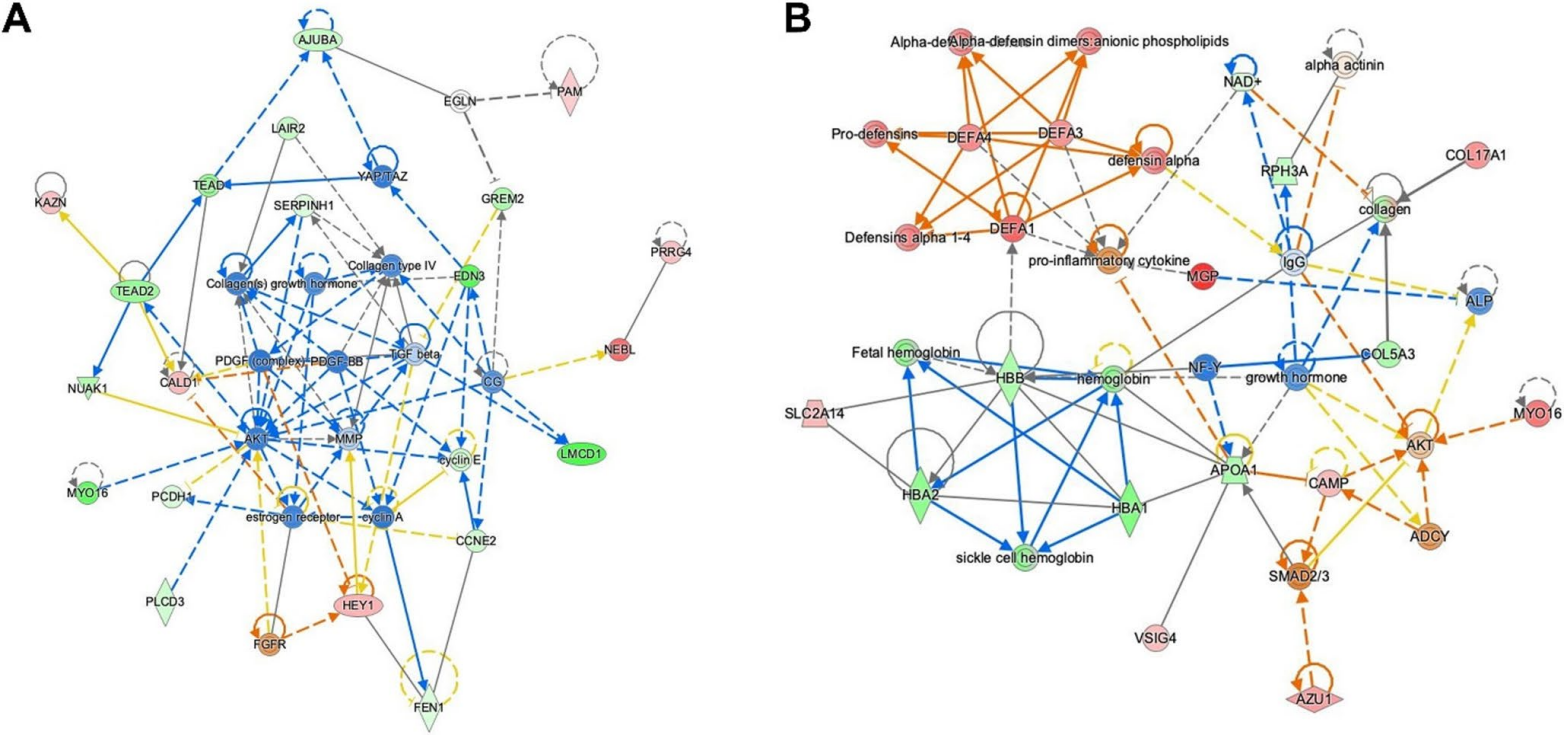
Significant networks from Ingenuity Pathway Analysis. **A** This top network was constructed using the DEGs identified in the symptomatic analysis. The network reflects dense signaling between collagens, TGF beta, and MMP. **B** This was the most significant network generated with the DEGs identified in the PHASES analysis. Many signaling pathways in this network are evident between defensins, cytokines, and the DEGs

We first examined differential expression for symptomatic vs. asymptomatic IAs. After removing genes with low expression and limiting to protein coding genes, we had a dataset of 13,348 genes. We identified 97 genes that met our criteria for differential expression (p-value < 0.01, fold-change > 2). Of these, 57 genes had lower expression in the symptomatic group and 40 had increased expression (Supplementary Table 4). No ontologies were associated with the decreased genes, but the genes with increased expression reflected responses to type II interferon, cytokine, peptide, and a defense response to other organism. In our analysis with IPA, macrophage classical activation signaling pathway was the only predicted activated pathway. There were 10 significant upstream regulators, 3 of which were inhibited. Multiple of the activated upstream regulators were cytokines, namely TNF, IFNA2, and IFNB1. In addition, there were 6 significant networks constructed based on connections with the DEG set. The top network reflects signaling centered around TGF-beta, collagens, and MMP as shown in Fig. 2A.

In the PHASES analysis, we began with a gene set of 13,177 protein coding genes. Here we identified 38 DEGs, 17 of which had lower expression in the high-risk group and 21 with increased expression (Supplementary Table 5). One gene, *MYO16*, was identified here and in the symptomatic analysis. The down regulated genes were associated with oxygen carrier activity. Biological processes associated with the genes with increased expression in the high-risk group included humoral immune response, cell killing, and organ or tissue specific immune response. Based on these DEGs, IPA identified multiple pathways that were predicted to be activated, which include neutrophil extracellular trap signaling, neutrophil degranulation, and S100 family signaling. No significant upstream regulators were associated with this set of differentially expressed genes. Lastly, there were 2 significant networks. The most significant network had associated functions of cardiovascular disease, cell death and survival, and connective tissue disorders. There were many defensins within this network, along with collagen and pro-inflammatory cytokine as shown in Fig. 2B.

### Principal component and clustering analyses

**Fig. 3:**
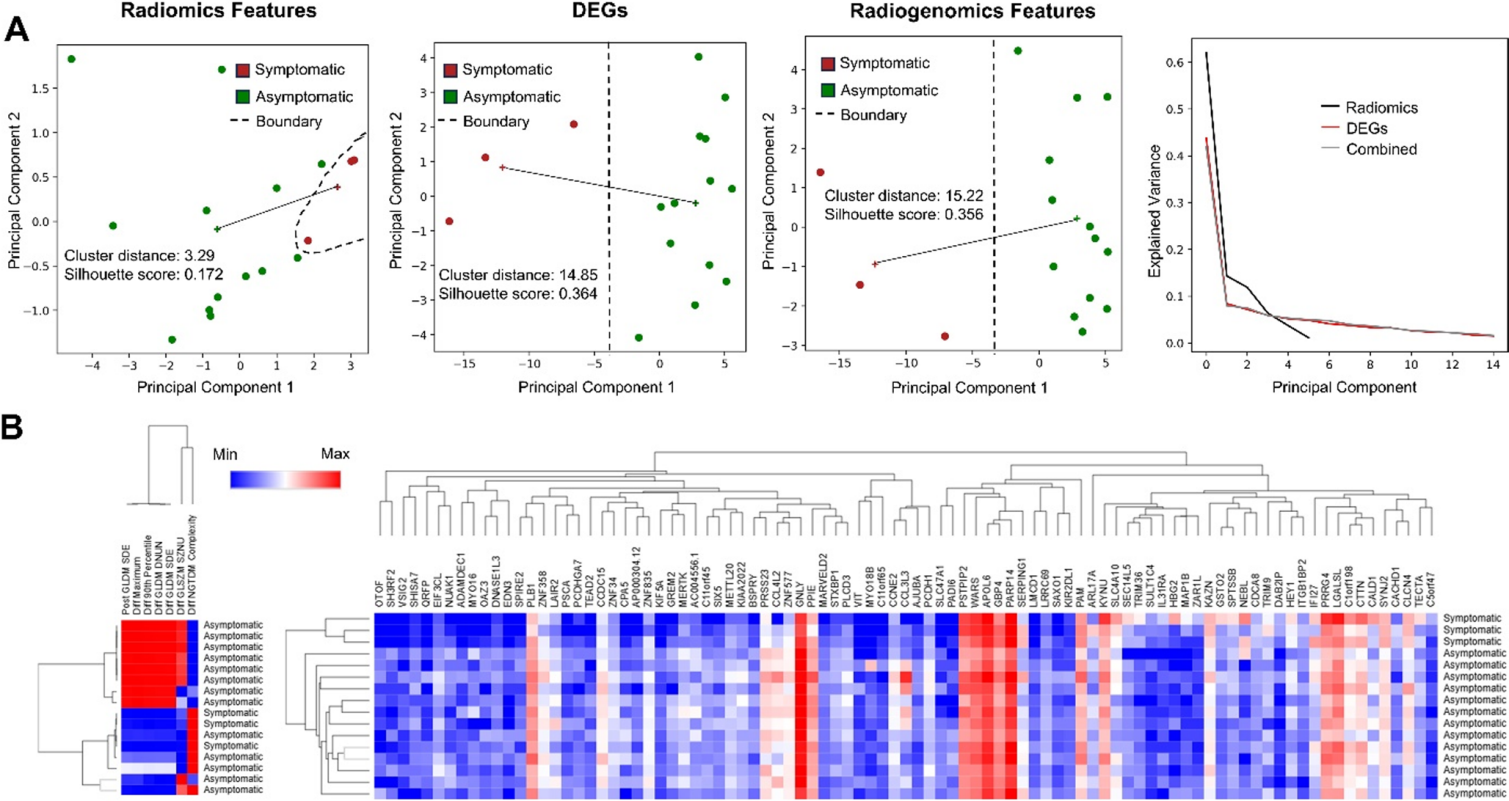
Analysis of symptomatic and asymptomatic IAs. **A** Principal component analysis of different feature sets (Radiomics, DEGs, and radiogenomics features) based on their symptomatic status. The + represents the cluster centroid and the solid line represents the inter-cluster distance. **B** Hierarchical clustering of all samples using radiomics and DEGs with respect to their symptomatic status

A total of 7 RFs and 91 DEGs were used for PCA analysis of symptomatic vs. asymptomatic IAs. We observed that RFs only, DEGs only, and radiogenomics features all had good separation as shown in Fig. 3A. We observed that the distance between the centroids of the symptomatic and asymptomatic clusters using RFs only was low (3.29) as compared to PCA performed with DEGs (14.85) and the radiogenomics features (15.22). Similarly, we also observed that the silhouette score was higher in the DEGs and radiogenomics as compared to the RFs only PCA (0.364 and 0.356 vs. 0.172 respectively). Through clustering analysis, we observed that 2 of the symptomatic IAs were clustered together using RFs only, whereas all the symptomatic IAs were in proximity while using DEGs only as shown in Fig. 3B.

**Fig. 4:**
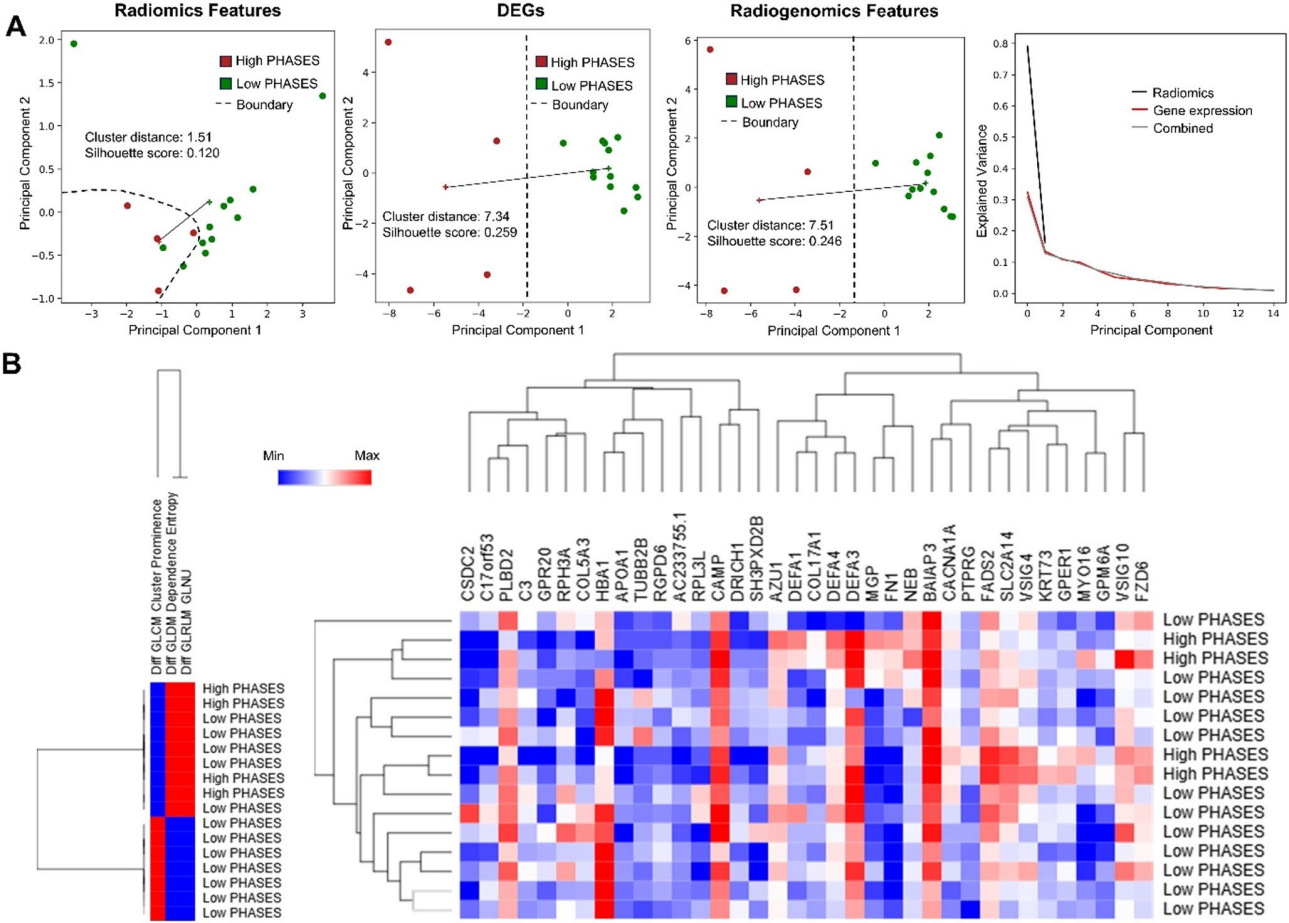
Analysis of high and low-risk IAs based on their PHASES score. **A** Principal component analysis of different feature sets (Radiomics, DEGs, and radiogenomics features) based on PHASES score. The + represents the cluster centroid and the solid line represents the inter-cluster distance. **B** Hierarchical clustering of all samples using radiomics and DEGs with respect to their PHASES score (PHASES ≥ 6 was considered high-risk)

In our PHASES based risk assessment, we used 3 RFs and 36 DEGs for PCA analysis. The separation between high-risk and low-risk IAs was moderate using RFs only; however, this improved significantly when using the DEGs and radiogenomics features as shown in Fig. 4A. We observed that the distance between the centroids of the high-risk and low-risk clusters using RFs only was low (1.51), as compared to that with DEGs (7.34) and the radiogenomics features (7.51). The silhouette score was higher in the DEGs and radiogenomics as compared to the RFs only PCA (0.259 and 0.246 vs. 0.120 respectively). Through clustering analysis, we observed that high-risk IAs were separated using RFs only and using DEGs only as well (Fig. 4B).

### Correlation between radiomics features and DEGs

**Fig. 5:**
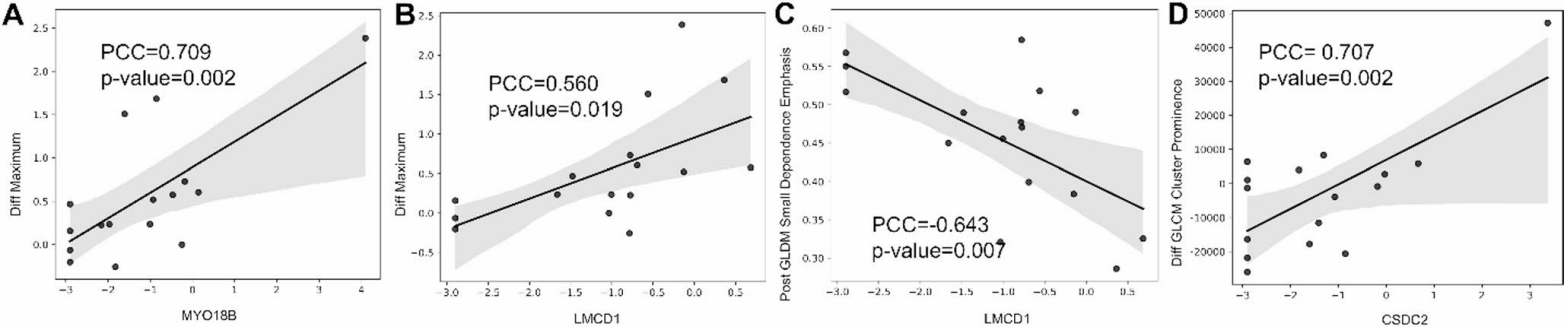
Correlation analysis of significant radiomics features and genes. Correlation plots of representative radiomics features and differentially expressed genes. The light gray region around the solid line represents the 95% confidence interval for the regression plot. A positive correlation was found between Diff. Maximum and MYO18B expression (**A**), and LMCD1 expression (**B**) while a negative correlation was observed for Post GLDM small dependence emphasis and LMCD1 expression (**C**). From the PHASES-based DEGs and RFs, Diff. cluster prominence was positively correlated with CSDC2 (**D**)

Through correlation analysis of significantly different RFs and DEGs using symptomatic status, we observed that there were 7 unique RFs that had correlations with 38 DEGs. *OTOF*, *MYO18B*, and *LMCD1* had the most correlates. Of note, we observed that the difference in maximum signal intensity was positively correlated with *MYO18B* gene (PCC = 0.709, *p* = 0.002) and *LMCD1* gene (PCC = 0.560, *p* = 0.019). We also observed that the GLDM small dependence emphasis from post-contrast MRI correlates negatively with *LMCD1* (PCC=−0.643, *p* = 0.007) as shown in Fig. 5. A complete list of all correlations between RFs and genes is given in Supplementary Table 6. Similarly, correlation analysis of the significantly different RFs and DEGs through PHASES based assessment revealed significant correlations between 3 unique RFs and 5 unique DEGs. Notably, we observed that there was a strong positive correlation between the GLCM cluster prominence in the difference between post and pre-contrast MRI and the *CSDC2* gene (PCC = 0.707, *p* = 0.002). A complete list of all correlates is given in Supplementary Table 7.

## Discussion

Aneurysm wall enhancement and differential gene expression analysis have individually been demonstrated as tools for robust risk stratification of IAs. In this pilot study, we combined the features obtained from both these modalities and investigated the effects on risk stratification of IAs. We observed that a total of 18 unique radiomics features and 128 unique genes were significantly different between high-risk and low-risk IAs. We also observed that DEGs had the highest variations and inter cluster distances in PCA and grouped well in hierarchical clustering. Finally, we also observed that there were 46 gene correlates to 10 radiomics features. To our knowledge, this is the first study evaluating a combined radiogenomics approach for risk stratification of IAs.

In univariate analysis of RFs, we observed that symptomatic IAs had a lower GLSZM small area emphasis and higher GLDM small dependence emphasis in post-contrast MRI. Lower GLSZM small area emphasis and higher GLDM small dependence emphasis are indicative of large size zones and coarse textures hence, these trends could indicate local heterogeneity in wall tissue composition due to inflammation and vasa vasorum. Similarly, when exploring radiomics differences through PHASES-based risk stratification, we observed high-risk IAs had a higher GLRLM non-uniformity which reflects a higher heterogeneity in post-contrast MRI. Additionally, a lower difference in GLSZM small area low gray level emphasis indicates that post contrast MRI had lower areas of low gray levels. These could be due to focal enhancement of tissue which could result in small patches of high signal intensity in the post-contrast MRI as compared to pre-contrast MRI.

Specifically examining the differentially expressed genes selected in the symptomatic analysis, we note several genes that are involved in critical biological processes, including immune response, cell signaling, and cell differentiation. *ADAMDEC1*, a secreted protein from the disintegrin metalloproteinase family, is associated with immune response, negative regulation of cell adhesions, and proteolysis. *EDN3*, which encodes member of the endothelin family, is a vasoactive peptide that influences an array of biological functions relevant in IA pathogenesis, such as leukocyte chemotaxis, cell proliferation, and smooth muscle contraction. Lastly, *IL31RA*, which encodes a protein belonging to the type I cytokine receptor family, is implicated in immune system processes, including cytokine-mediate signaling, macrophage and monocyte differentiation, and regulation of both apoptotic and proliferative processes. Using IPA, we identified enrichment of interferon signaling, and the macrophage classical activation signaling pathway, which facilitates macrophage-driven inflammation and immune responses. IPA also found TNF signaling, another critical pathway in inflammation and immune responses, to be associated with these genes, suggesting how active these processes are in high-risk IAs.

Focusing on genes identified as differentially expressed between IAs with low and high PHASES score, we again find genes associated with critical processes in IA pathogenesis, such as immune responses, extracellular matrix (ECM) organization, and wound healing. *CAMP* encodes a protein that is crucial for chemotaxis, immune mediator induction, and regulation of the inflammatory response, particularly in chronic inflammation. Further, it plays an active role in defense responses, neutrophil activation, and the positive regulation of angiogenesis and cell proliferation. Defensins, a family of antimicrobial and cytotoxic peptides, are abundant in neutrophil granules and are involved in immune response and chemotaxis. *COL17A1* and *COL5A3* are both key structural constituents of the extracellular matrix, contributing to cell-matrix adhesion and ECM organization. Lastly, *FN1* encodes fibronectin, another key ECM component, supporting angiogenesis, cell-matrix adhesion, and the regulation of cell migration and chemotaxis. The key themes we identified with the complete set of DEGs through IPA include neutrophil degranulation and extracellular traps (NETs), which have been linked to IA rupture [27]. We also note this gene set reflects structural activity, evidenced by collagens and *FN1*, and inflammation, seen through cytokine and NFkB signaling.

From the correlation analysis, we observed that *MYO18B* was significantly correlated with the difference in maximum intensity between the pre- and post-contrast scans. *MYO18B* is a gene that is associated with cell adhesion and migration and had a higher expression in symptomatic IAs in our analysis. A higher degree could lead to disruption in endothelial function thus allowing selective transport of contrast particles into IA wall thus leading to greater enhancement, which is measured by the difference in maximum intensity between pre- and post-contrast MRI. We also observe a positive correlation of *LMCD1* gene with the difference in maximum intensity between pre- and post-contrast MRI which quantifies enhancement and a negative correlation with GLDM small dependence emphasis from post-contrast MRI which quantifies heterogeneity in texture. *LMCD1* gene is associated with thrombin-induced *IL-33* expression and migration which in turn play a role in endothelial dysfunction [28]. A higher degree of endothelial dysfunction could result in homogeneous proliferation and deposition of contrast particles in the IA wall thus leading to a heterogeneous enhancement of the wall. Finally, through PHASES-based DEGs and RFs, we observed CSDC2 was positively correlated with Diff. cluster prominence. CSDC2 is a protein binding gene that binds to 3’ UTRs of mRNAs like histone H3.3 which could influence inflammatory conditions leading to higher heterogeneous AWE quantified by GLCM cluster prominence.

Accurate risk assessment of newly discovered IAs is paramount for timely management and intervention. Multiple studies have shown that AWE can be used for accurate risk assessment of IAs as well as to gain pathobiological insights onto local wall pathobiology [8, 29]. Most recently, Sagues et al. demonstrated that adding AWE to existing clinical and morphological characteristics improves risk stratification of IAs [30]. However, a major drawback is the lack of specificity where many low risk IAs are also flagged as high risk due to presence of AWE [11]. Complementary risk assessment tools for IAs include blood-based biomarkers which use transcriptomics from RNA sequencing of blood for risk stratification of IAs. Past studies by our group have demonstrated the potential of transcriptomic based models that use circulating blood to identify intracranial aneurysms [31–34]. We have additionally identified transcripts associated with IA risk as assessed by multiple clinical metrics, namely IA size, PHASES, and predicted aneurysm trajectory or PAT score, and used them to build predictive models [16, 35]. For our previous analysis of IA risk using PHASES, we identified 76 differentially expressed genes (q-value < 0.05, fold-change > 2) in a cohort of 68 samples collected from individuals with IA, 26 of which were considered high-risk. A linear discriminant analysis (LDA) model based on these transcripts achieved a testing accuracy of 88%. Furthermore, multiple transcripts, including *COL17A1* and members of the defensin family, were identified in the original study and this current study. In this study, we demonstrate that the synergistic combination of both modalities can aid in better risk stratification. This could indeed be used as a clinical tool for rapid risk stratification [36]. Future multi-center studies based on large datasets can use the methodology presented herein to develop robust IA risk stratification pipelines.

There are multiple limitations associated with this study. The predominant limitation is the small and imbalanced sample size. It is challenging to identify patients who have undergone AWE and whole blood RNA sequencing. We continue to enroll patients and are collaborating with other centers, so that we can repeat the study in a larger cohort in the future to validate our findings here. Secondly, none of the DEGs were significantly different after false discovery rate correction based on PHASES score. Therefore, in this small, preliminary exploration we selected features based on unadjusted p-values. The potential correlations and mechanistic pathways suggested are also limited by the small sample size analyzed in this study and future studies with more robust datasets are warranted for better confidence. Thirdly, the correlations presented are based purely on the correlation coefficient and causal nature of these correlations need to be validated rigorously in more comprehensive histopathological studies with larger sample sizes. Lastly, symptomatic status and PHASES may not reflect true rupture risk but act as surrogates for this endpoint.

## Conclusion

In this study, we used an established pipeline to assess AWE and genomics analyses to characterize differences between high and low-risk intracranial aneurysms. We observed that a radiogenomics approach, which combines genomics and MRI-based radiomics data, can help improve risk stratification of IAs. We also observed high degrees of correlation between genomics and radiomics features. While this study was conducted in a limited number of cases, these findings demonstrate the potential utility of radiogenomics in improving risk stratification of IAs.

## Electronic supplementary material

Below is the link to the electronic supplementary material.

Supplementary Material 1

## Data Availability

Data is available from corresponding author upon reasonable request.
