# Supplementary material for: Evaluation of radiogenomics for risk stratification of intracranial aneurysms: a pilot study
Source: Neuroradiology. 2025 Jul 15;67(9):2425–35. doi: 10.1007/s00234-025-03702-1 (PMC12546524; doi:10.1007/s00234-025-03702-1)
Supplement: Supplementary file 1 — Supplementary Material 1 [file 234_2025_3702_MOESM1_ESM.docx]

**Supplementary Tables**

|  | **Asymptomatic (n=13)** | **Symptomatic (n=3)** | **p-value** | **Low-Risk**  **PHASES (n=12)** | **High-Risk**  **PHASES (n=4)** | **p-value** |
| --- | --- | --- | --- | --- | --- | --- |
| Age | 69.08±6.65 | 56.67±11.26 | 0.069 | 65.5±9.78 | 70.5±5.22 | 0.292 |
| Sex | 9 (69%) | 2 (67%) | 1.0 | 8 (67%) | 3 (75%) | 1.0 |
| Smoking | 7 (54%) | 2 (67%) | 1.0 | 6 (50%) | 3 (75%) | 0.771 |
| Hypertension | 11 (85%) | 1 (33%) | 0.267 | 9 (75%) | 3 (75%) | 1.0 |
| Familial history of aneurysm | 2 (15%) | 1 (33%) | 1.0 | 3 (25%) | 0 (0%) | 0.712 |
| Size (mm) | 3.89±1.92 | 2.9±1.11 | 0.089 | 3.09±0.92 | 5.54±2.53 | 0.051 |
| Size Ratio | 1.33±0.75 | 1.12±0.42 | 0.5 | 1.08±0.36 | 1.94±1.03 | 0.151 |
| Aspect Ratio | 1.01±0.45 | 0.81±0.12 | 0.343 | 0.84±0.24 | 1.37±0.56 | **0.039** |
| ICA | 3 (23%) | 0 (0%) | 0.918 | 3 (25%) | 0 (0%) | 0.712 |
| MCA | 3 (23%) | 1 (33%) | 1.0 | 2 (17%) | 2 (50%) | 0.505 |
| AComm/Posterior | 7 (54%) | 2 (67%) | 1.0 | 7 (58%) | 2 (50%) | 1.0 |
| AWE | 2 (15%) | 0 (0%) | 1.0 | 1 (8%) | 1 (25%) | 1.0 |

**Supplementary Table 1.** Demographics of patients with symptomatic and asymptomatic IAs as well as low-risk and high-risk IAs based on PHASES.

ICA: Internal carotid artery; MCA: Middle cerebral artery; AComm: Anterior communicating artery; AWE: Aneurysm wall enhancement

**Supplementary Table 2.** Significantly different radiomics features between symptomatic and asymptomatic IAs.

| **Feature** | **Asymptomatic** | **Symptomatic** | **p-value** | **q-value** | **Hedge’s G** |
| --- | --- | --- | --- | --- | --- |
| Diff GLDM Small Dependence Emphasis | -0.045±0.039 | 0.019±0.015 | 0.014 | 0.039 | 1.657 |
| Diff GLSZM Zone Percentage | -0.046±0.045 | 0.019±0.024 | 0.025 | 0.039 | 1.448 |
| Diff NGTDM Complexity | -112.337±143.438 | 114.86±77.887 | 0.025 | 0.039 | 1.579 |
| Post GLDM Small Dependence Emphasis | 0.435±0.087 | 0.545±0.026 | 0.039 | 0.039 | 1.289 |
| Post GLSZM Size Zone NonUniformity Normalized | 0.423±0.073 | 0.514±0.033 | 0.039 | 0.039 | 1.254 |
| Post GLSZM Small Area Emphasis | 0.671±0.061 | 0.743±0.023 | 0.039 | 0.039 | 1.201 |
| Post GLSZM Zone Percentage | 0.523±0.094 | 0.639±0.024 | 0.039 | 0.039 | 1.253 |
| Diff First Order 90Percentile | 0.293±0.554 | -0.153±0.073 | 0.039 | 0.039 | 0.82 |
| Diff First Order Maximum | 0.685±0.743 | -0.036±0.182 | 0.039 | 0.039 | 0.987 |
| Diff First Order Range | 0.675±0.634 | -0.108±0.187 | 0.039 | 0.039 | 1.253 |
| Diff GLDM Dependence NonUniformity Normalized | -0.029±0.027 | 0.015±0.025 | 0.039 | 0.039 | 1.541 |
| Diff GLSZM Size Zone NonUniformity | -20.343±27.489 | 10.513±15.154 | 0.039 | 0.039 | 1.118 |

Post: Radiomics based on Post-contrast MRI; Diff: Radiomics based on difference between Pre- and Post-contrast MRI.

**Supplementary Table 3.** Significantly different radiomics features between high-risk and low-risk IAs based on PHASES.

| **Feature** | **Low PHASES** | **High PHASES** | **p-value** | **q-value** | **Hedge’s G** |
| --- | --- | --- | --- | --- | --- |
| Diff GLCM Cluster Prominence | 1711.792±17200.743 | -16364.549±10811.822 | 0.03 | 0.042 | 1.065 |
| Diff GLCM Cluster Tendency | 3.181±44.899 | -33.255±13.605 | 0.042 | 0.042 | 0.855 |
| Diff GLCM Imc1 | -0.007±0.035 | 0.034±0.022 | 0.03 | 0.042 | 1.174 |
| Diff GLDM Dependence Entropy | 0.037±0.178 | -0.129±0.066 | 0.042 | 0.042 | 0.977 |
| Diff GLRLM Gray Level NonUniformity | 2.974±8.921 | 5.847±5.165 | 0.042 | 0.042 | 0.329 |
| Diff GLSZM Gray Level NonUniformity | -0.209±1.557 | 2.228±1.16 | 0.013 | 0.042 | 1.556 |

Diff: Radiomics based on difference between Pre- and Post-contrast MRI.

**Supplementary Table 4.** Differentially expressed genes between symptomatic and symptomatic IAs.

| **Genes** | **p-value** | **q-value** | **log(Fold Change)** | **Hedge’s G** |
| --- | --- | --- | --- | --- |
| KIF5A | <0.001 | 0.007 | -5.924 | 3.705 |
| WARS | <0.001 | 0.007 | 1.568 | 1.844 |
| LMCD1 | <0.001 | 0.007 | -6.162 | 3.486 |
| SYNJ2 | <0.001 | 0.012 | 1.241 | 2.457 |
| SLC47A1 | <0.001 | 0.038 | -6.387 | 2.083 |
| ADAMDEC1 | <0.001 | 0.038 | -6.065 | 2.433 |
| HBG2 | <0.001 | 0.043 | 4.191 | 1.248 |
| SPIRE2 | <0.001 | 0.043 | -3.839 | 2.956 |
| KYNU | <0.001 | 0.052 | 1.155 | 1.647 |
| GSTO2 | <0.001 | 0.067 | 2.13 | 1.829 |
| C11orf45 | <0.001 | 0.067 | -2.559 | 3.281 |
| SEC14L5 | <0.001 | 0.067 | 2.019 | 1.634 |
| CCNE2 | <0.001 | 0.067 | -1.65 | 2.991 |
| CPA5 | <0.001 | 0.083 | -5.676 | 1.826 |
| APOL6 | <0.001 | 0.094 | 1.161 | 1.631 |
| PSTPIP2 | <0.001 | 0.112 | 1.49 | 1.276 |
| MYO16 | <0.001 | 0.139 | -5.324 | 1.895 |
| KAZN | <0.001 | 0.168 | 1.268 | 1.808 |
| VSIG2 | <0.001 | 0.168 | -5.454 | 1.684 |
| VIT | <0.001 | 0.168 | -5.799 | 1.598 |
| SLC4A10 | <0.001 | 0.168 | 1.77 | 1.706 |
| QRFP | <0.001 | 0.168 | -5.861 | 1.555 |
| PRRG4 | <0.001 | 0.184 | 1.244 | 1.648 |
| EIF3CL | <0.001 | 0.184 | -2.256 | 2.538 |
| ARL17A | <0.001 | 0.184 | 2.082 | 0.648 |
| OTOF | <0.001 | 0.184 | -6.074 | 1.476 |
| PPIE | <0.001 | 0.19 | -1.043 | 2.22 |
| PLB1 | <0.001 | 0.196 | -1.102 | 2.202 |
| STAT1 | <0.001 | 0.196 | 1.184 | 1.522 |
| TRIM36 | <0.001 | 0.196 | 2.128 | 1.594 |
| DAB2IP | <0.001 | 0.196 | 2.714 | 2.365 |
| SH3RF2 | <0.001 | 0.213 | -5.983 | 1.428 |
| SULT1C4 | <0.001 | 0.238 | 2.213 | 2.189 |
| MYO18B | <0.001 | 0.247 | -7.642 | 1.278 |
| SHISA7 | <0.001 | 0.302 | -2.685 | 1.672 |
| TEAD2 | <0.001 | 0.335 | -3.584 | 1.872 |
| CCL4L2 | 0.001 | 0.355 | -2.525 | 2.284 |
| SIX5 | 0.001 | 0.382 | -2.309 | 2.223 |
| OAZ3 | 0.001 | 0.405 | -3.794 | 1.647 |
| DNASE1L3 | 0.001 | 0.405 | -1.881 | 2.694 |
| BSPRY | 0.001 | 0.405 | -2.462 | 2.123 |
| IFI27 | 0.001 | 0.417 | 1.576 | 1.324 |
| CCDC15 | 0.002 | 0.442 | -1.096 | 2.042 |
| LAIR2 | 0.002 | 0.442 | -1.96 | 1.835 |
| HEY1 | 0.002 | 0.442 | 1.502 | 1.747 |
| ZNF34 | 0.002 | 0.442 | -1.202 | 2.675 |
| EDN3 | 0.002 | 0.453 | -5.744 | 1.192 |
| CCL3L3 | 0.002 | 0.493 | -4.75 | 1.565 |
| AP000304.12 | 0.002 | 0.521 | -2.738 | 1.88 |
| PARP9 | 0.002 | 0.521 | 1.063 | 1.368 |
| PSCA | 0.002 | 0.521 | -2.733 | 2.024 |
| TRIM9 | 0.002 | 0.551 | 1.941 | 1.195 |
| LGALSL | 0.002 | 0.551 | 1.047 | 1.461 |
| SERPINH1 | 0.003 | 0.558 | -1.236 | 1.998 |
| MARVELD2 | 0.003 | 0.558 | -1.904 | 2.644 |
| MAP1B | 0.003 | 0.558 | 2.446 | 1.259 |
| PARP14 | 0.003 | 0.563 | 1.206 | 1.317 |
| C1orf198 | 0.003 | 0.579 | 1.024 | 1.694 |
| STXBP1 | 0.003 | 0.579 | -1.819 | 1.972 |
| GBP1 | 0.003 | 0.586 | 1.279 | 1.282 |
| GREM2 | 0.003 | 0.586 | -2.92 | 1.669 |
| ZNF577 | 0.003 | 0.608 | -1.05 | 2.093 |
| PAM | 0.004 | 0.646 | 1.025 | 1.263 |
| LRRC69 | 0.004 | 0.646 | -1.28 | 2.311 |
| C11orf65 | 0.004 | 0.656 | -3.057 | 1.789 |
| FEN1 | 0.004 | 0.665 | -1.196 | 2.47 |
| SAXO1 | 0.004 | 0.665 | -2.376 | 2.231 |
| AC004556.1 | 0.005 | 0.678 | -2.129 | 1.489 |
| IL31RA | 0.005 | 0.719 | 1.802 | 1.595 |
| CTTN | 0.005 | 0.731 | 1.009 | 1.459 |
| PRSS23 | 0.006 | 0.731 | -1.165 | 1.417 |
| PCDH1 | 0.005 | 0.731 | -1.291 | 1.953 |
| TECTA | 0.006 | 0.744 | 1.241 | 1.291 |
| RPP40 | 0.006 | 0.744 | -1.021 | 2.321 |
| CALD1 | 0.006 | 0.744 | 1.414 | 0.971 |
| NEBL | 0.006 | 0.796 | 2.949 | 1.807 |
| CACHD1 | 0.006 | 0.8 | 1.65 | 1.195 |
| ZNF358 | 0.007 | 0.821 | -1.007 | 1.745 |
| GNLY | 0.007 | 0.821 | -1.161 | 1.544 |
| PCDHGA7 | 0.007 | 0.821 | -2.019 | 1.538 |
| PADI6 | 0.007 | 0.852 | -3.838 | 1.218 |
| CDCA8 | 0.007 | 0.856 | 1.087 | 1.791 |
| KIR2DL1 | 0.008 | 0.859 | -1.774 | 1.883 |
| SPTSSB | 0.008 | 0.863 | 1.222 | 1.282 |
| GBP4 | 0.008 | 0.867 | 1.003 | 0.948 |
| SERPING1 | 0.008 | 0.867 | 1.506 | 0.965 |
| METTL20 | 0.009 | 0.867 | -1.146 | 2.051 |
| ZAR1L | 0.008 | 0.867 | 1.697 | 1.168 |
| AJUBA | 0.009 | 0.867 | -2 | 1.796 |
| PLCD3 | 0.008 | 0.867 | -1.592 | 1.818 |
| ZNF835 | 0.009 | 0.867 | -1.354 | 1.766 |
| KIAA2022 | 0.009 | 0.867 | -1.483 | 1.663 |
| MERTK | 0.009 | 0.872 | -1.592 | 1.669 |
| CLCN4 | 0.009 | 0.879 | 1.15 | 1.369 |
| ITGB1BP2 | 0.009 | 0.879 | 1.419 | 1.359 |
| C5orf47 | 0.01 | 0.882 | 1.858 | 1.306 |
| NUAK1 | 0.01 | 0.89 | -2.727 | 1.482 |

**Supplementary Table 5.** Differentially expressed genes between low-risk and high-risk IAs based on PHASES.

| **Genes** | **p-value** | **q-value** | **log(Fold Change)** | **Hedge’s G** |  |
| --- | --- | --- | --- | --- | --- |
| BAIAP3 | <0.001 | 0.612 | 1.284 | 1.65 | |
| CSDC2 | <0.001 | 0.612 | -7.214 | 1.295 | |
| MGP | <0.001 | 0.825 | 3.694 | 0.544 | |
| DEFA1 | <0.001 | 0.825 | 2.748 | 0.967 | |
| HBA1 | <0.001 | 0.882 | -3.911 | 1.511 | |
| C17orf53 | <0.001 | 0.882 | -3.053 | 2.389 | |
| CAMP | <0.001 | 0.882 | 1.267 | 1.334 | |
| DEFA4 | <0.001 | 0.882 | 2.098 | 0.94 | |
| GPR20 | <0.001 | 0.882 | -2.928 | 1.797 | |
| AZU1 | <0.001 | 0.949 | 1.716 | 0.842 | |
| COL17A1 | 0.002 | 1 | 1.891 | 1.473 | |
| HBB | 0.005 | 1 | -2.563 | 1.229 | |
| FADS2 | 0.001 | 1 | 1.189 | 1.033 | |
| APOA1 | 0.001 | 1 | -2.653 | 1.893 | |
| SLC2A14 | 0.008 | 1 | 1.256 | 0.902 | |
| KRT73 | 0.01 | 1 | 1.147 | 1.339 | |
| RPH3A | 0.009 | 1 | -1.956 | 0.94 | |
| PLBD2 | 0.004 | 1 | -1.019 | 1.811 | |
| VSIG10 | 0.003 | 1 | 1.128 | 1.367 | |
| MYO16 | 0.001 | 1 | 2.422 | 1.615 | |
| AC233755.1 | 0.009 | 1 | -1.694 | 1.536 | |
| HBA2 | 0.002 | 1 | -3.493 | 1.529 | |
| RPL3L | 0.008 | 1 | -2.417 | 0.985 | |
| C3 | 0.008 | 1 | -1.411 | 1.882 | |
| COL5A3 | 0.008 | 1 | -2.744 | 1.275 | |
| CACNA1A | 0.007 | 1 | 1.245 | 1.191 | |
| RGPD6 | 0.005 | 1 | -1.781 | 1.435 | |
| NEB | 0.008 | 1 | 1.449 | 0.815 | |
| FN1 | 0.009 | 1 | 3.252 | 0.666 | |
| DRICH1 | 0.002 | 1 | -2.122 | 1.755 | |
| PTPRG | 0.009 | 1 | 1.255 | 1.277 | |
| GPM6A | 0.005 | 1 | 1.474 | 1.671 | |
| SH3PXD2B | 0.005 | 1 | -2.693 | 1.253 | |
| TUBB2B | 0.01 | 1 | -2.611 | 0.848 | |
| GPER1 | 0.008 | 1 | 1.2 | 1.105 | |
| DEFA3 | 0.009 | 1 | 1.907 | 0.816 | |
| FZD6 | 0.006 | 1 | 1.055 | 1.675 | |
| VSIG4 | 0.008 | 1 | 1.147 | 0.934 | |

**Supplementary Table 6**: Correlation between differentially expressed genes and radiomics features using symptomatic status.

| **Feature** | **Gene** | **PCC** | **p-value** |
| --- | --- | --- | --- |
| Diff First order 90Percentile | MYO18B | 0.774 | <0.001 |
| Diff First order Maximum | MYO18B | 0.709 | 0.002 |
| Diff GLSZM Size Zone NonUniformity | LMCD1 | -0.691 | 0.003 |
| Diff First order Maximum | OTOF | 0.685 | 0.003 |
| Diff NGTDM Complexity | KIF5A | -0.684 | 0.003 |
| Diff GLDM Dependence NonUniformity Normalized | NUAK1 | -0.657 | 0.006 |
| Diff NGTDM Complexity | DAB2IP | 0.649 | 0.007 |
| Post GLDM Small Dependence Emphasis | LRRC69 | -0.646 | 0.007 |
| Post GLDM Small Dependence Emphasis | LMCD1 | -0.643 | 0.007 |
| Diff GLDM Small Dependence Emphasis | NUAK1 | -0.641 | 0.007 |
| Diff NGTDM Complexity | MERTK | -0.624 | 0.01 |
| Diff GLDM Small Dependence Emphasis | LMCD1 | -0.62 | 0.01 |
| Diff NGTDM Complexity | C11orf65 | -0.615 | 0.011 |
| Diff NGTDM Complexity | NUAK1 | -0.613 | 0.012 |
| Diff GLDM Small Dependence Emphasis | C11orf45 | -0.611 | 0.012 |
| Diff GLSZM Size Zone NonUniformity | LRRC69 | -0.596 | 0.015 |
| Diff NGTDM Complexity | SPIRE2 | -0.592 | 0.016 |
| Diff NGTDM Complexity | GNLY | -0.584 | 0.017 |
| Diff First order Maximum | LMCD1 | 0.58 | 0.019 |
| Diff NGTDM Complexity | HEY1 | 0.579 | 0.019 |
| Diff NGTDM Complexity | PCDHGA7 | -0.571 | 0.021 |
| Post GLDM Small Dependence Emphasis | ADAMDEC1 | -0.57 | 0.021 |
| Diff GLDM Dependence NonUniformity Normalized | OTOF | -0.562 | 0.024 |
| Diff GLDM Small Dependence Emphasis | OTOF | -0.554 | 0.026 |
| Diff GLDM Dependence NonUniformity Normalized | LAIR2 | -0.553 | 0.026 |
| Diff NGTDM Complexity | SIX5 | -0.553 | 0.026 |
| Diff GLDM Small Dependence Emphasis | VIT | -0.553 | 0.026 |
| Diff GLDM Small Dependence Emphasis | PCDH1 | -0.55 | 0.027 |
| Diff GLDM Dependence NonUniformity Normalized | GNLY | -0.549 | 0.028 |
| Diff First order 90Percentile | ZNF358 | 0.548 | 0.028 |
| Diff NGTDM Complexity | C11orf45 | -0.547 | 0.028 |
| Diff GLDM Small Dependence Emphasis | CCL4L2 | -0.543 | 0.03 |
| Diff First order Maximum | KIAA2022 | 0.542 | 0.03 |
| Diff First order 90Percentile | CLCN4 | -0.54 | 0.031 |
| Diff GLDM Small Dependence Emphasis | METTL20 | -0.535 | 0.033 |
| Diff GLDM Small Dependence Emphasis | MYO18B | -0.534 | 0.033 |
| Diff NGTDM Complexity | GSTO2 | 0.534 | 0.033 |
| Diff NGTDM Complexity | ZNF577 | -0.533 | 0.034 |
| Diff GLDM Dependence NonUniformity Normalized | KIF5A | -0.532 | 0.034 |
| Diff GLDM Dependence NonUniformity Normalized | C11orf45 | -0.532 | 0.034 |
| Diff GLSZM Size Zone NonUniformity | PADI6 | -0.532 | 0.034 |
| Diff GLDM Small Dependence Emphasis | ZNF577 | -0.532 | 0.034 |
| Diff First order 90Percentile | OTOF | 0.53 | 0.035 |
| Post GLDM Small Dependence Emphasis | KIR2DL1 | -0.52 | 0.039 |
| Diff GLSZM Size Zone NonUniformity | PCDHGA7 | -0.519 | 0.04 |
| Diff GLDM Small Dependence Emphasis | PSCA | -0.517 | 0.04 |
| Diff GLDM Dependence NonUniformity Normalized | CCL4L2 | -0.514 | 0.042 |
| Post GLDM Small Dependence Emphasis | C5orf47 | 0.512 | 0.043 |
| Post GLDM Small Dependence Emphasis | SLC47A1 | -0.51 | 0.044 |
| Diff GLDM Small Dependence Emphasis | SH3RF2 | -0.509 | 0.044 |
| Diff NGTDM Complexity | OTOF | -0.508 | 0.044 |
| Diff GLDM Small Dependence Emphasis | SPIRE2 | -0.508 | 0.045 |
| Diff NGTDM Complexity | OAZ3 | -0.507 | 0.045 |
| Diff NGTDM Complexity | GREM2 | -0.507 | 0.045 |
| Diff GLDM Small Dependence Emphasis | PRSS23 | -0.507 | 0.045 |
| Diff GLDM Small Dependence Emphasis | SLC47A1 | -0.506 | 0.045 |
| Diff NGTDM Complexity | MYO18B | -0.503 | 0.047 |
| Diff GLDM Small Dependence Emphasis | PCDHGA7 | -0.501 | 0.048 |
| Post GLDM Small Dependence Emphasis | MYO16 | -0.5 | 0.049 |
| Diff GLSZM Size Zone NonUniformity | IL31RA | 0.5 | 0.049 |
| Diff GLDM Dependence NonUniformity Normalized | MYO18B | -0.5 | 0.049 |
| Diff GLDM Small Dependence Emphasis | EIF3CL | -0.499 | 0.049 |
| Diff GLDM Dependence NonUniformity Normalized | PRSS23 | -0.498 | 0.05 |

Post: Radiomics based on Post-contrast MRI; Diff: Radiomics based on difference between Pre- and Post-contrast MRI.

**Supplementary Table 7**: Correlation between differentially expressed genes and radiomics features using PHASES score.

| **Feature** | **Gene** | **PCC** | **p-value** |
| --- | --- | --- | --- |
| Diff GLCM Cluster Prominence | CSDC2 | 0.707 | 0.002 |
| Diff GLDM Dependence Entropy | CSDC2 | 0.589 | 0.016 |
| Diff GLCM Cluster Prominence | C17orf53 | 0.576 | 0.019 |
| Diff GLRLM Gray Level NonUniformity | KRT73 | 0.54 | 0.031 |
| Diff GLDM Dependence Entropy | RPL3L | 0.508 | 0.044 |
| Diff GLDM Dependence Entropy | APOA1 | 0.503 | 0.047 |
| Diff GLDM Dependence Entropy | C17orf53 | 0.503 | 0.047 |

Diff: Radiomics based on difference between Pre- and Post-contrast MRI.
